# Wearable Sensor-Based Human Activity Recognition Method with Multi-Features Extracted from Hilbert-Huang Transform

**DOI:** 10.3390/s16122048

**Published:** 2016-12-02

**Authors:** Huile Xu, Jinyi Liu, Haibo Hu, Yi Zhang

**Affiliations:** 1 Department of Automation, Tsinghua National Laboratory for Information Science and Technology (TNList), Tsinghua University, Beijing 100084, China; hl-xu16@mails.tsinghua.edu.cn (H.X.); zhyi@tsinghua.edu.cn (Y.Z.); 2 Key Laboratory of Dependable Service Computing in Cyber Physical Society Ministry of Education, Chongqing University, Chongqing 400044, China; 3 School of Computer and Control, University of Chinese Academy of Sciences, Beijing 100190, China; liujinyi16@mails.ucas.ac.cn; 4 School of Software Engineering, Chongqing University, Chongqing 401331, China

**Keywords:** activity recognition, Hilbert-Huang transform, feature extraction, wearable sensors

## Abstract

Wearable sensors-based human activity recognition introduces many useful applications and services in health care, rehabilitation training, elderly monitoring and many other areas of human interaction. Existing works in this field mainly focus on recognizing activities by using traditional features extracted from Fourier transform (FT) or wavelet transform (WT). However, these signal processing approaches are suitable for a linear signal but not for a nonlinear signal. In this paper, we investigate the characteristics of the Hilbert-Huang transform (HHT) for dealing with activity data with properties such as nonlinearity and non-stationarity. A multi-features extraction method based on HHT is then proposed to improve the effect of activity recognition. The extracted multi-features include instantaneous amplitude (IA) and instantaneous frequency (IF) by means of empirical mode decomposition (EMD), as well as instantaneous energy density (IE) and marginal spectrum (MS) derived from Hilbert spectral analysis. Experimental studies are performed to verify the proposed approach by using the PAMAP2 dataset from the University of California, Irvine for wearable sensors-based activity recognition. Moreover, the effect of combining multi-features vs. a single-feature are investigated and discussed in the scenario of a dependent subject. The experimental results show that multi-features combination can further improve the performance measures. Finally, we test the effect of multi-features combination in the scenario of an independent subject. Our experimental results show that we achieve four performance indexes: recall, precision, F-measure, and accuracy to 0.9337, 0.9417, 0.9353, and 0.9377 respectively, which are all better than the achievements of related works.

## 1. Introduction

In recent years, services provided by computing devices have shifted from proprietary computing services to flexible services focusing on human need. Different kinds of small devices with computing power and communications capabilities have grown in popularity. Computer systems are closely linked to human users by natural interaction methods. Thus, how to recognize human activities is an important part of supporting technology for these computer systems.

At present, two of the major identification methods are based on vision sensors and wearable sensors. Identification methods, which are based on computer vision, use images taken with cameras, and these images are intuitive and understandable. Many public places are provided with 24-h video monitoring equipment, providing a rich source of data for gesture recognition. Meanwhile, as an often-considered subject in computer science, computer vision technology has accumulated plenty of research results. However, since the human body is particular, and activity is ambiguous, diversiform, and different in space and time, it is difficult to get a higher recognition rate with computer vision-based methods. At the same time, there are some limitations of the camera. For example, the use of cameras is limited by factors such as light conditions and install locations. Alternatively, due to the rapid development of electronic technology, physical sensors have the characteristics of small dimensions, high precision, and low cost. So, activity recognition based on wearable sensors has become more and more popular.

Currently, wearable sensors-based human activity recognition plays an important role in disease prevention, rehabilitative training, daily health monitoring, and many other fields. With respect to disease prevention, Sazonov et al. [1] propose a novel methodology for automatic recognition of postures and activities in patients with stroke by a wearable shoe-based device. Iosifidis et al. [2] extend the independent living of older persons in the early stages of dementia by identifying human eating and drinking activities. Amft et al. [3] use on-body sensors for automatic dietary monitoring aimed at lowering the health risks for many chronic diseases, including obesity. In the field of rehabilitative training, it has been pointed out that we can apply automatic recognition of human activities in monitoring the daily life of elderly people [4], fall detection for the elderly [5] and motor symptoms in Parkinson’s disease [6]. In daily healthy monitoring, Zhu et al. apply a wearable system in the constant monitoring of physiological parameters of infants such as heart rate, pulse, invasive blood pressure, and early gastric cancer (EGC) [7]. In addition, as the universal value for a healthy life, automatic classification of daily activities is used for the promotion of health-enhancing physical activities and a healthier lifestyle [8].

Most of the existing works utilize mean value, variance, fast Fourier transform (FFT), and wavelet transform (WT) as feature extraction tools in a wearable sensors-based activity recognition system. The methods described above are applied to signals which have great linearity and stationarity. However, the output signal of wearable sensors manifest properties such as nonlinearity and non-stationarity [9]. If we use simple filtering operations, such as band-pass or low-pass filtering, sometimes we may destroy sharp features of movement patterns in the activity signals. Thus, Hilbert-Huang transform (HHT) is introduced to solve these problems for dealing with nonlinear and non-stationary signals. At this point, state-of-the-art works [9,10,11] have discussed the application of empirical mode decomposition (EMD) in activity recognition. In these works, all the intrinsic mode functions (IMFs) or part of them are selected as features. These features were then combined with other time-domain features to derive some statistics from them. However, these works do not consider using the Hilbert spectral analysis to further process these IMFs and instead use the physical quantities from Hilbert spectral analysis as inputs for the classifier.

According to a further investigation, we find that when we apply features extracted from EMD to activity recognition, there are still some manifest weaknesses. The classification results obtained from extracted features by EMD or combinations of EMD and other non-HHT approaches are significantly dependent on the type of activities. That is to say, by using these features for classification, the performance matrix shows remarkable fluctuation among different kinds of human activities (such as walking, lying, running, etc.). Therefore, we propose that some other useful features of HHT should be explored and utilized for activity recognition to improve the effect of classification. Therefore, in this paper, we present a multi-features extraction method based on HHT to improve the effect of activity recognition. The extracted multi-features include IA and IF obtained from EMD, as well as IE and MS derived from Hilbert spectral analysis. To evaluate the proposed approach, we perform two groups of experiments by using the physical activity monitoring for aging people dataset 2 (PAMAP2) as input signals and utilizing the back propagation (BP) neural network as a classifier. The effect of combining multi-features vs. a single-feature are investigated and discussed in the scenario of a dependent subject. The experimental results show that combined multi-features instantaneous energy density, marginal spectrum, instantaneous amplitude, and instantaneous frequency (IE-MS-IA-IF) can further improve the performance. Finally, we test the effect of multi-features combination in the scenario of an independent subject. The experimental results show that we achieve four performance indexes: recall, precision, F-measure, and accuracy to 0.9337, 0.9417, 0.9353, and 0.9377 respectively, which are all better than related works tested on the same dataset.

The rest of this paper is structured as follows. In Section 2, an extensive review of feature extraction and classification algorithm is presented. Section 3 introduces the public dataset and data preprocessing method we use in this study, and Section 4 describes the activity recognition methodology in detail and analyzes the degree of stationarity (DS) for activity data. In Section 5, the classification methods and parameters setting of BP neural network are introduced. Next, we present the results obtained from experiments and compare the performance measures with relevant work in Section 6. Then, the limitation and future work are discussed in Section 7. Finally, the paper is summarized in Section 8.

## 2. Related Work

The algorithm for the wearable sensors activity recognition system contains two parts, feature extraction and activity classification. In this section, we will introduce some common methods for these two parts.

### 2.1. Feature Extraction

In the time-domain, a signal’s characteristics contain the statistical properties of the data collected from the tri-axial accelerometers such as mean value, mean square root, and variance. The mean value and the mean square root are the expression of the signal’s magnitude, and the other statistical properties reflect the volatility and dispersion of the data. Some time ago, Li et al. proposed a shapelet-based model which can effectively recognize complex activities [12] in the time domain.

The space-domain characteristics describe correlation coefficient for different sensors on different body parts. Frequency domain characteristics include the entropy and the energy of acceleration data. They are generally used as a measurement of the periodicity of a piece of data. Some of the existing works perform activity recognition using frequency characteristic from short-time Fourier transform (STFT) [13], Wigner-Ville distribution(WVD), and WT [14,15].

HHT is a novel signal analysis theory proposed by Huang in the 1990s [16]. The main innovative point of HHT is that it proposes the idea of intrinsic mode function (IMF) and introduces the EMD method. HHT decomposes raw data to several IMFs by using the EMD method and obtains IF by using the Hilbert transform (HT) on each IMF. At that point, signals are finally expressed as energy distribution in the time-frequency domain, this is called the Hilbert spectrum. We can also further get the MS of the signal by the method proposed in [16]. HHT is designed to work for data that is non-stationary and nonlinear [17], such as the propagation of seismic waves [18,19] and detection of earthquake’s hypocenter [20,21].

In addition, HHT is also used for analysis of other nonlinear systems such as atomic decomposition [22], biomedicine [23], earthquake engineering [24], structural damage monitoring [25], radar data processing [26], mechanical equipment fault diagnosis [27], and diffusion of contaminants [28]. For activity recognition, Wang et al. [10], and Liao et al. [11] introduced EMD to process accelerometer signals to extract features such as IA and IF. Ayachi et al. [9] developed algorithms for automatic detection and segmentation of activities of daily living (ADL) tasks, by using a combination of EMD and other transform approaches. However, to the best of our knowledge, for activity recognition, little work discusses and evaluates the effects of these combined features to classification. Moreover, although it is one of the major components, Hilbert spectral analysis is yet to be introduced to feature extraction in the field of activity recognition.

### 2.2. Classifier for Activity Recognition

Some classification algorithms in human activity recognition are commonly used at present, such as naive Bayes, decision tree, support vector machine (SVM), and K-nearest neighbor (KNN). This subsection will explain these algorithms briefly.

Naive Bayes is a simple classifier which is widely used in human activity recognition [29,30,31,32]. In naive Bayes, a posteriori probability is obtained from priori probability according to new input. The advantage of naive Bayes is that it only requires a small amount of training data to estimate the parameters necessary for classification. Meanwhile, the disadvantage is that we need to know a priori probability in advance.

Decision tree is also a widely adopted algorithm in this field [29,30]. The strength of it is that the decision-making process is intuitive and well-understood [33]. Yet, when many values are uncertain or outcomes are linked, the calculation can be very complex, and even lead to overfitting.

KNN is a non-parametric method used for classification and regressions [12]. The advantage of KNN is that it is simple to implement and far less expensive to retrain, but it leads to a large amount of computations when there are a great many training samples [34].

SVM is a kind of pattern recognition method which is based on statistical theory [35]. SVM has the advantage of high classification accuracy and does not need a large number of samples to input, thus, SVM is widely used in activity recognition [36,37]. However, a potential drawback of the SVM is that the input data require full labeling of input data.

Compared with the above algorithms, BP neural networks have strong fault-tolerance, robustness, and self-learning ability. So, we choose the BP neural network as the classifier in this paper.

## 3. Dataset and Signal Preprocessing

This section presents the dataset and signal preprocessing method we use in our paper. In order to contrast with existing work, the public dataset called PAMAP2 is used. It was contributed by the University of California, Irvine [38]. It was donated on 6 August 2012.

### 3.1. Hardware Setup

The dataset PAMAP2 uses three inertial measurement units (IMUs) and one heart rate monitor as sensors during the data collection. Each IMU contains two 3-aixs micro-electro-mechanical system (MEMS) accelerometers, one scale is ±16 g and the other is ±6 g, one $\pm 1500^{\circ }$/s 3-axis MEMS gyroscope, and one 3-axis magneto-resistive magnetic sensor whose scale is ±400 μT. All IMUs were sampled at 100 Hz, and the heart rate monitor was sampled at 9 Hz [39]. One IMU and heart rate monitor was on the chest, one IMU over the wrist on the dominant arm, and the other IMU on the dominant side’s ankle.

The previous work [40] shows that three sensors can achieve a great balance between recognition accuracy and numbers of sensors. In wearable sensors systems, as the number of sensors increases, the average recognition accuracy increases, but this is neither comfortable nor practical for users. Nevertheless, previous work showed that one or two sensors are not enough for real-time activity recognition [41]. Thus, in this paper, we use the accelerometer data from three sensors.

### 3.2. Subject and Data Format

There were eight males and one female who participated in the data collection. Their age ranged from 23.91 to 30.53 years, body mass index (BMI) ranged from 22.49 kg·m${}^{-2}$ to 27.73 kg·m${}^{-2}$, one subject was left-handed and the other eight were right-handed. In accordance with the regulation, they all collected over 10 h of data separately.

The dataset PAMAP2 contains 12 activities (defined in Table 1), including basic activities (running, walking, cycling, and nordic walking), postures (lying, sitting, and standing), everyday work (ascending stairs and descending stairs), household (ironing and vacuum cleaning), and motor movements (rope jumping). Each subject had to follow this protocol. Beyond that, if the subject had additional free time, they were allowed to collect six optional activities to enrich the range of the dataset, the activities were watching TV, computer work, car driving, folding laundry, house cleaning, and playing soccer.

### 3.3. Data Processing Flow

**Figure 1:**
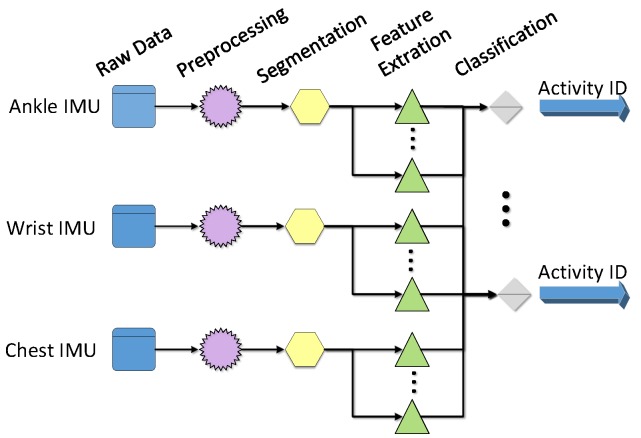
Data processing flow diagram. IMU: inertial measurement unit.

This section provides a brief introduction to the data processing flow in this paper. The process diagram is shown in Figure 1.

The data processing flow, including data preprocessing, data segmentation, feature extraction, and classification.

### 3.4. Data Preprocessing

The real-time data received was saved as text-files, 54 columns in each text-file. Each IMU’s data contain the columns as laid out in Table 2 and the meaning of each column is shown in Table 3.

Data labeled with activity ID = 0 mainly covers transient activities between performing different activities. We discard them first. At the same time, to ensure the accuracy of each activity, we discard the first 10 s of the data. As we can see in Table 3, columns 14–17 are invalid in this data collection. Then, to avoid repeating the calculation and according to the recommendation of the dataset, we choose to use ±16 g 3D-accelerometer data. That is to say, we eliminated the data in columns 5–7 and 14–17.

### 3.5. Data Segmentation

Wearable activity recognition systems are composed of a set of sensors worn on the human body, and the sensors deliver a data stream every second. To accurately recognize each period of activity, we should deliver the data stream to each activity period every time. Since we use neural networks as a classifier in this work, we also need to consider the number of activity periods we delivered each time. In this section, we present the data segment method we use in our paper-sliding window approach.

The sliding window approach is one of the most popularly used segmentation methods, and is based on the thinking that the real-time data is more valuable than the stale data [42]. So, in the field of activity recognition, the sliding window approach has been widely employed in previous works [43,44]. However, in the segmentation process, how to choose a window size is one of the key problems. Decreasing the window size allows a faster activity detection, and reduces resources and energy needs, however, it may not contain one complete activity cycle. On the contrary, large data windows are normally considered for the recognition of complex activities but will result in the increment of computational cost. A range of window sizes from 0.08 s [45] to 6.7 s [30], or even 30 s [44] have been used in previous studies. The sliding window approach has been proven to be especially beneficial for the recognition of periodic signal. However, different activities have different periods. It is hard to determine the best window size for one dataset.

In this paper, we use the dataset from Reiss and Stricker [39]. For comparision with their work, we use a similar preprocessing method. The preprocessed data is segmented using a sliding window with 5.12 s of window size, shifted by 1 s between consecutive windows.

## 4. The Feature Extraction Method Based on HHT

It is known to us that only when processed data has stationarity, linearity, and periodicity, can those data be converted to frequency domain by FT. Then, the frequency characteristic can be obtained. The data processed by WT can be non-stationary, but it is required to be linear. However, most of the data we meet in real life is nonlinear. What is worse, only a few of them can be approximately seen as linear data. So, both WT and FT have their own limitations. To overcome the above limitation, Huang proposed HHT [16] to process a nonlinear and non-stationary signal, which has been proven to be effective in practical engineering. Besides, HHT presents the results of feature extraction in time-frequency-energy space and the results contain physical meaning, which makes it easier for us to understand. In this section, we will briefly introduce the HHT algorithm and analyze the stationarity of the activity data.

### 4.1. A Brief Introduction to HHT

The steps of HHT algorithm are shown as follows and the detailed introduction to each step is described in the corresponding subsection.

Step 1 EMD. The EMD method is needed to decompose the raw signal into several IMFs to which the HT can be applied. Then the raw signal can be denoted by the sum of IMFs and residue.
Step 2 HT. Using the HT extend the real signal IMFs into the complex plane. Next, the IA and IF can be derived based on the result of HT.
Step 3 Hilbert spectral analysis. The Hilbert spectrum can be deduced according to the relationship among IF, IA and time. The final result of this step is to show the frequency-time distribution of signal amplitude and we can analyze the localized features of signal from it.
Step 4 The derivation of IE, MS, and DS. Based on the Hilbert spectrum, we can get the results of IE, MS, and DS. Furthermore, we can analyze the characteristics and stationarity of different activity data.

### 4.2. EMD

According to [16], if we directly use HT to convert activity data, the obtained IF may be negative. That is to say, the IF will lose physical meaning. To avoid this situation, HHT decomposes raw data to several IMFs by using the EMD method. IMF needs to have two characteristics:

- In the whole dataset, the number of extrema and the number of zero-crossings must be equal or differ by no more than one.
- At any time points, the average value of the envelope defined by the local maxima and the local minima equals zero.

**Figure 2:**
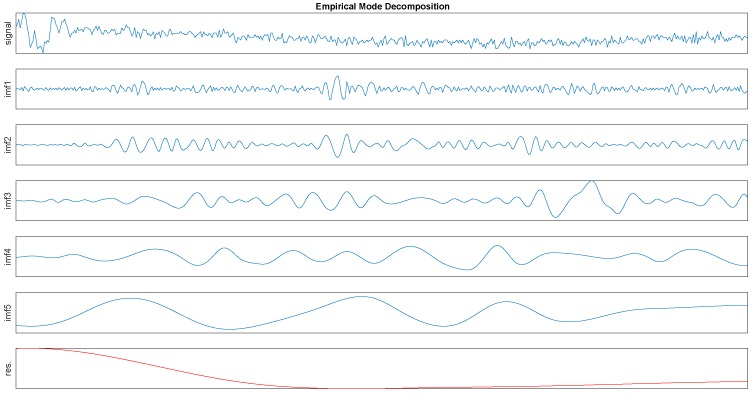
The empirical mode decomposition (EMD) components from the activity data. Notice that the first one is the raw data and the last component plotted by red line is a trend instead of an IMF.

In order to give a intuitive impression first, the EMD process is shown in Figure 2. Then we will give a detailed interpretation of this algorithm as follows.

We identify all extrema of a signal, then use cubic spline interpolation to connect all the local maxima and repeat this method to process all the local minima. The constructed upper and lower envelops will cover all the data between them. The mean of these two envelops is calculated and denoted as $m_{1}$. Besides, a new component $h_{1}$ can be obtained from the difference between the raw data and $m_{1}$, denoted by

(1) $$X\left(t\right)-m_{1}=h_{1}$$

However, in reality, $h_{1}$ is always not an IMF because of the overshoots and undershoots, which can generate new extrema and influence this process. Thus, in order to get a better result, the sifting process will be repeated several times. In the second sifting process, the result of the first sifting process $h_{1}$ serves as the raw data, then we repeat above steps until the result can be regarded as an IMF. The first IMF component can be designated as

(2) $$c_{1}=h_{1k}$$

where $c_{1}$ is the first IMF component and the *k* means the sifting times.

Moreover, we can eliminate $c_{1}$ from the raw data and designate the rest of the data as

(3) $$X\left(t\right)-c_{1}=r_{1}$$

Then we regard the $r_{1}$ as the raw data and repeat using the above sifting process. This mentioned process is iterative and IMFs $c_{2},\ldots ,c_{n}$ can be obtained. The whole algorithm will stop until the residue is too small to continue or it becomes a monotonic function from which no more IMF can be extracted. Finally, the raw data X(t) can be denoted by

(4) $$X\left(t\right)=\sum_{i=1}^{n}c_{i}+r_{n}$$

where the X(t) is the raw signal, $c_{i}$ is the ith IMF and $r_{n}$ is the residue. The EMD algorithm (Algorithm 1) also can be expressed as follows.

**Algorithm 1** EMD algorithm**Input:** The raw data X(t), i=1
**Output:** The IMFs $c_{1},\ldots ,c_{n}$, and the residue $r_{n}$
1:**Subfunction** for IMF component2:**loop**3:  Using cubic spline interpolation to respectively connect all the local maxima and minima.4:  Calculate the mean of these two envelops and denote it as *m*.5:  A new component h=X(t)−m6:  **if** the new component *h* is a IMF **then**7:      C=h8:  **else**9:      X(t)=h and repeat the loop.10:  **end** **if**11:**end** **loop**12:**End Subfunction**13:  14:**Main function**15:call **Subfunction**16:The rest of the data $r_{i}=X\left(t\right)-C$17:**while** the $r_{i}$ do not meet the stop criteria **do**18:  i++19:  $X\left(t\right)=r_{i}$ and call **Subfunction**20:  $c_{i}=C$21:  $r_{i}=r_{i-1}-C$22:**end** **while**23:**End Main function**

### 4.3. HT

The HT of a signal is the same as the convolution of a signal with the function $h\left(t\right)=\frac{1}{\pi t}$. In order to get the instantaneous features of activity data, we use the HT to convert each IMF component:

(5) $$y\left(t\right)=H\left(x\left(t\right)\right)=\frac{1}{\pi }\int_{\infty }^{-\infty }\frac{x(\tau )}{t-\tau }\;d\tau \;$$

where x(t) indicates the IMF that will be converted, y(t) is the result after transform. Next, z(t) can be derived on this basis.

(6) z(t)=x(t)+iy(t)

Moreover, the IA a(t) and instantaneous phase θ(t) about z(t) can be obtained.

(7) $$a\left(t\right)=\sqrt{x^{2}\left(t\right)+y^{2}\left(t\right)}$$

(8) $$\theta \left(t\right)=arctan\frac{y(t)}{x(t)}$$

The IF ω(t) is defined as the derivative of instantaneous phase θ(t).

(9) $$\omega \left(t\right)=\frac{d\theta (t)}{dt}$$

### 4.4. Hilbert Spectral Analysis

**Figure 3:**
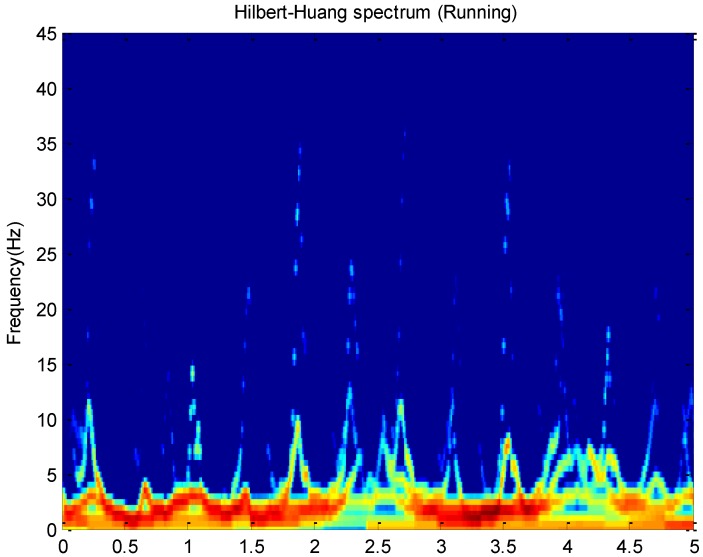
The Hilbert-Huang spectrum for the running data with 100 Hz frequency. The activity energy appears in skeleton lines representing each intrinsic mode function (IMF). The Hilbert-Huang spectrum was filtered by a 9×9 weighted Gaussian filter.

**Figure 4:**
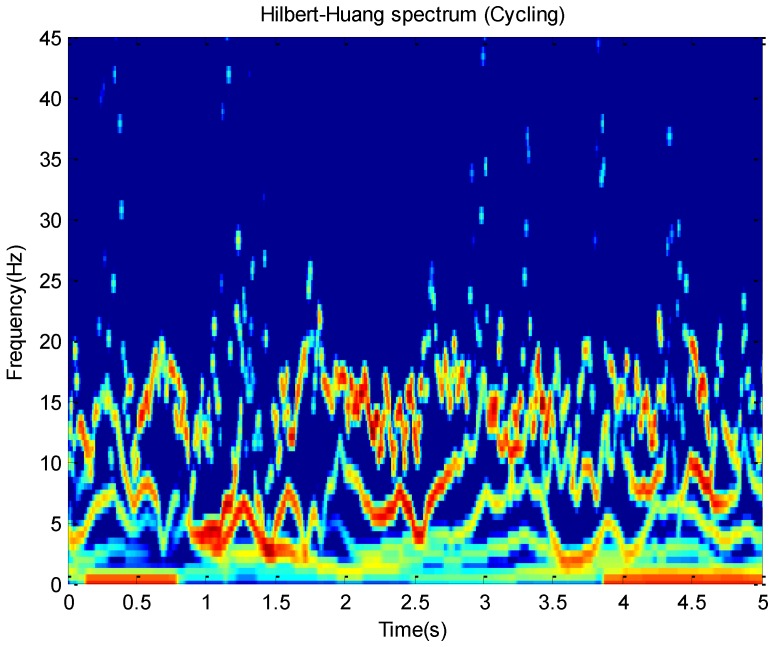
The Hilbert-Huang spectrum for the cycling data with 100 Hz frequency. The energy distribution of skeleton lines gives a better comparison with other types of activities. The Hilbert-Huang spectrum was filtered by a 9×9 weighted Gaussian filter.

The Hilbert spectrum H(ω,t) can be deduced according to the relationship among IF ω(t), IA a(t) and time. For example, this paper plots Hilbert spectrums about running and cycling in Figure 3 and Figure 4. These activity data all come from the *x*-axis of the accelerometer on the wrist.

The Hilbert spectrum in the colour map format for the activity data is given in Figure 3 and Figure 4. The energy of each IMF was plotted as a skeleton line. In order to give a intuitive feeling on IE, the Hilbert spectrum in the smoothed format is obtained by using 9×9 weighted Gaussian filter. As we can see from the above figure, the energy of the activity running is concentrated mainly in the 0–5 Hz frequency region while the energy of activity cycling is dispersedly distribute in 0–25 Hz frequency region. This can reflect the exercise intensity of these activities. At the same time, some features about energy can be deduced.

### 4.5. IE and MS

Based on the Hilbert spectrum H(ω,t), we can get definitions about MS and IE. Then, we denote them as h(ω) and IE(t) respectively.

(10) $$h\left(\omega \right)=\int_{0}^{T}H\left(\omega ,t\right)dt$$

(11) $$IE\left(t\right)=\int_{\omega }H^{2}\left(\omega ,t\right)d\omega$$

**Figure 5:**
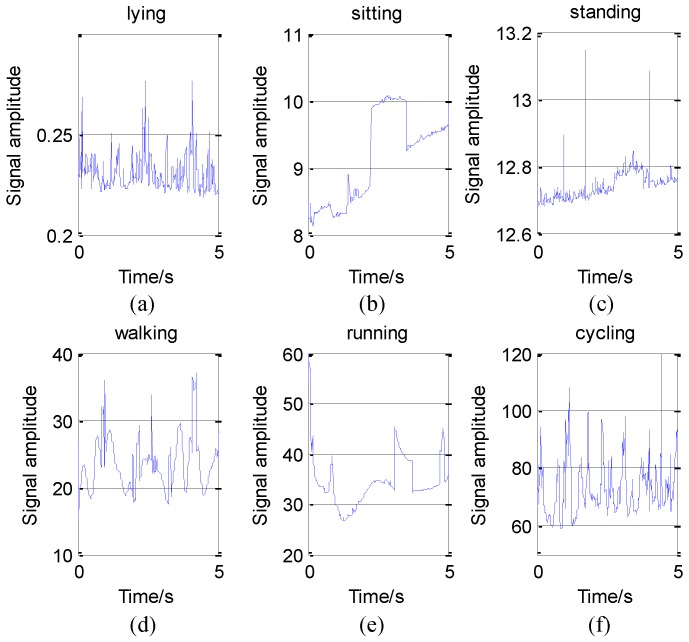
The instantaneous energy density level (IE) of six different activities. (**a**) The IE of lying data, the signal amplitude is very small; (**b**,**c**) The IE of sitting and standing, which are similar in signal amplitude; (**d**–**f**) The IE of walking, running, and cycling respectively, their amplitude and variation range of them are much larger. All data come from the z-axis of the accelerometer on the wrist.

According to the above analysis, we can use HHT to convert each piece of data and get their IE. These activity data all come from the *z*-axis of the accelerometer on the wrist. The IE of activity 1 to activity 6 is shown in Figure 5.

As we can see in Figure 5, in some time periods, the numerical value and variation range of IE differ from activity to activity. On one hand, from the view of numerical value, the IE of lying is around 0.2 when the IE of cycling is around 80. On the other hand, from the view of variation range, the peak-to-peak value of lying only 0.1. If we plot lying and cycling in one figure, we will find that the numerical value of lying is nearly a constant.

On the contrary, the peak-to-peak value of cycling can be more than 100. That is to say, cycling is more drastic than lying, which conforms to our intuitive feeling. Meanwhile, it also conforms to Huang’s theory that physical quantities such as IF, MS, and IE gained by HHT have practical meanings. So, we think HHT is more suitable for activity recognition.

**Figure 6:**
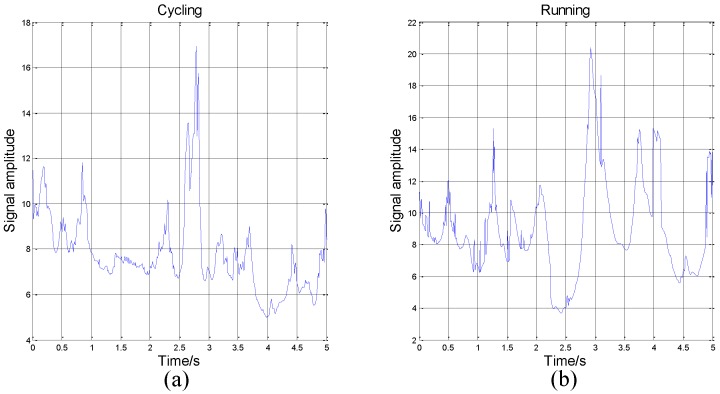
Two similar IE of two different types of activities. (**a**) The IE of cycling data; (**b**) The IE of running data. All data come from the z-axis of the accelerometer on the wrist.

However, in some time periods, the IE of two activities can be similar in numerical value and variation range. In this paper, we use the mean and the variance of IE as eigenvalues. The trend of signals’ amplitude may be different, but the mean and variance of IE can be same. So, if we only use one sensor to recognize activity, the judge result may be wrong. The comparison is shown in Figure 6.

In the athletic process, the exercise intensity will decrease when the participant feels tired. This will cause the feature of the activity to be similar to the features of other activities, which will affect the results of machine judgement. However, different body parts have different reactions during movement. The reaction of the same body part in different activities is similar, but we can solve the problem by adding sensors. Thus, this paper analyzes activity data from 3D accelerometers on the chest, wrist, and ankle. For an example, we consider IE as a feature, according to the above analysis, we can get the mean and the variance of IE to classify activities. Then, in total, we can get 18 eigenvalues from three sensors (each sensor has 6 eigenvalues, the mean and the variance of IE in x, y, z axis).

**Figure 7:**
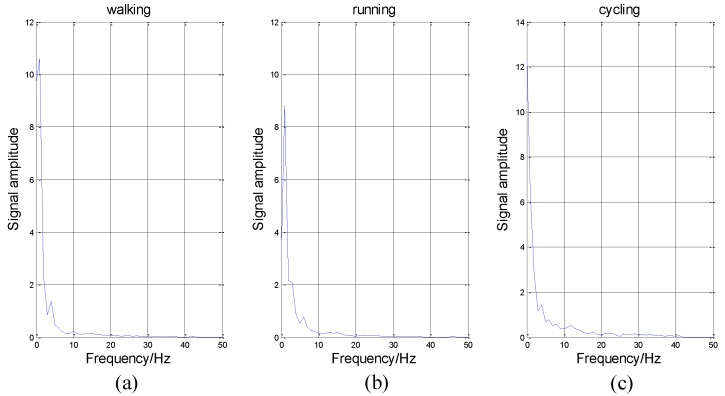
The marginal spectrum (MS) of three different activities. In some time periods, the variation tendency and signal amplitude of MS for different activities are similar. (**a**) The MS of walking data; (**b**) The MS of running data; (**c**) The MS of cycling data. All data come from the x-axis of the accelerometer on the wrist.

In addition to IE, there are many other features, such as IF, IA, and MS. We introduce them briefly. Firstly, MS represents the value of energy in each frequency. As shown in Figure 7, the abscissa axis represents frequency and the vertical axis represents energy intensity.

As shown in Figure 7, the variation tendency of these curves is similar. The energy in low frequency is much larger than that in high frequency, which conforms to our intuitive feeling that these activities are low-frequency-actions. It is impossible for people to perform these actions hundreds of times per second. Thus, in Figure 7, the energy is nearly equal to zero in high frequency.

The IF and the IA can greatly represent the instantaneous features of a signal. However, for one signal, we can get hundreds of (and even thousands of) eigenvalues about the IF and the IA. It will be difficult for the neural network to train such a large classification network. At the same time, it will need to spend lots of time finishing this work. Therefore, we also consider to use the mean and variance of them in our classification.

### 4.6. DS

This paper has mentioned several signal processing methods, including FT, STFT, and FFT which all require that the processed signal be stationary. However, this requirement is often ignored by researchers for simplification. Meanwhile, it is extremely difficult to meet the original definition of stationary signal. If we use that definition to judge a signal, nearly all signals will be considered as non-stationary signals.

So, Huang proposes a different definition for stationarity based on HHT [16]:

(12) $$n\left(\omega \right)=\frac{1}{T}h\left(\omega \right)$$

(13) $$DS\left(\omega \right)=\frac{1}{T}\int_{0}^{T}{\left(1-\frac{H(\omega ,t)}{n(\omega )}\right)}^{2}dt$$

Among them, n(ω) is the mean of the MS, DS(ω) is the DS of a signal.

If a signal is stationary, the Hilbert spectrum H(ω,t) will not be a function of time, and the DS(ω) will be zero. On the contrary, the higher the index value DS(ω) is, the more non-stationary the signal is. We analyze the data coming from the accelerometer located on the wrist as an example.

**Figure 8:**
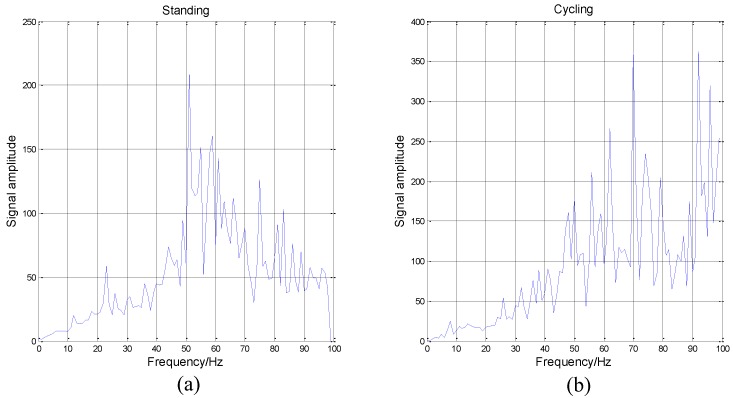
Degree of stationarity (DS) of two different types of activities. (**a**) The DS of standing data; (**b**) The DS of cycling data. All data come from the x-axis of the accelerometer on the wrist. The ordinate value represents the degree of non-stationarity of the data.

It can be seen from Figure 8 that the cycling data is highly non-stationary. The DS will increase when the frequency increases. Besides, the data of standing is the most non-stationary when the frequency is between 50 Hz to 60 Hz. Then, the overall DS of standing is also poor. Because of these obvious differences among different activities and activity data’s high non-stationarity, we determine that HHT is suitable for activity recognition.

## 5. Activity Classification

The above extracted features will be used as the inputs to this part, that is, the classification. Artificial neural networks are important methods of classification in the field of activity recognition, as proven by the work in [46,47,48]. Thus, in our work, the BP neural network is employed as the classifier to validate the extracted features.

### 5.1. Artificial Neural Network

A BP neural network is a multi-layers feed-forward neural network. According to statistics, 80% to 90% of the neural network models use the BP network or its extensions. Meanwhile, they are often used as a classifier in the field of activity recognition [49].

**Figure 9:**
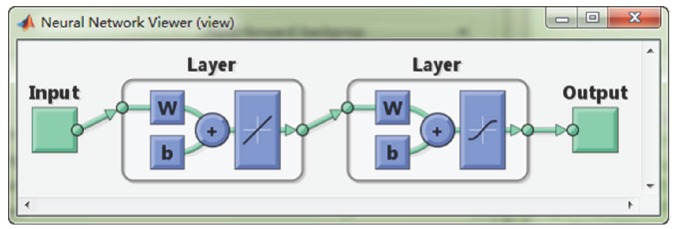
Schematic diagram of a three-layer neural network.

BP neural networks were proposed by Rumelhart and Mcclelland in 1986 [50]. From the aspect of structure, it is a typical multi-layers forward neural network with one input layer, several hidden layers, and an output layer. The full connection mode is adopted between the layers and a mutual connection does not exist between neurons in the same layer. The main characteristics of the BP neural networks are signal forward transmission, error backward propagation, and supervised learning. Moreover, it has been theoretically proven that a three-layer network with one hidden layer can approximate any nonlinear function [51]. A typical three-layer BP neural network is shown in Figure 9.

### 5.2. The Learning Algorithm Based on a BP Neural Network [52]

As shown in Figure 9 above, the structure of a three-layer BP neural network is demonstrated. The specific implementation steps of this supervised algorithm are given. The situation of more than one hidden layer can be derived based on this algorithm. Hereby, the BP neural network algorithm is shown as follows.

Step 1 Network initialization. The initial parameters of network should be set. For example, setting the weights of the input layer to the hidden layer, the hidden layer to the output layer as any small random number, and setting the initial threshold value.

Step 2 Training samples collection. According to the rules of supervised learning, both the input vector $X=(x_{1},x_{2},\cdots ,x_{m})$ and the corresponding output vector $D=(d_{1},d_{2},\cdots ,d_{l})$ should be provided.

Step 3 Output calculation. Calculating the output from the input layer to the hidden layer and the output layer.

For each neuron in the input layer: the input is $x_{i}$, the output is $O_{i}=x_{i}\left(i=1,2,\cdots ,m\right)$, *i* is the total number of neurons in the input layer.

For each neuron in the hidden layer: the input is $x_{j}=\sum_{i=1}^{m}w_{ij}O_{i}-\theta_{j}$, the output is $O_{j}=f\left(x_{j}\right)\left(j=1,2,\cdots \;n\right)$, *j* is the total number of neurons in the hidden layer.

For each neuron in the output layer: the input is $x_{k}=\sum_{j=1}^{m}w_{jk}O_{j}-\theta_{k}$, the output is $y_{k}=g\left(x_{k}\right)\left(k=1,2,\cdots \;n\right)$, *k* is the total number of neurons in the output layer.

In the above equations, $w_{ij}$ is the weight from the *i*th input layer to the *j*th hidden layer. $w_{jk}$ is the weight from the *j*th hidden layer to the *k*th output layer. f(x) and g(k) are transfer functions. There are many kinds of transfer functions which can be selected.

Step 4 Weight adjustment. According to the error, the BP neural network adjusts the weights from the output layer nodes to the hidden layer nodes and the weights from the hidden layer nodes to the input layer nodes. The network error function is defined as

(14) $$E=\frac{1}{2}\sum_{k=1}^{l}{\left(d_{k}-y_{k}\right)}^{2}$$

(15) w(t+1)=w(t)+(−η∇E(t))

In Equation (15), −∇E(t) represents inverse direction of the gradient of the error function in the *T*th training.

For the weight $w_{jk}\left(t+1\right)$ from the output layer to the hidden layer, the adjusting formula is

(16) $$w_{jk}\left(t+1\right)=w_{jk}\left(t\right)+\eta y_{k}\left(1-y_{k}\right)\left(d_{k}-y_{k}\right)O_{j}^{{}^{\prime }}$$

In Equation (16), *η* is the learning rate, η>0, *d* is the desired output.

For the weight $w_{ij}\left(t+1\right)$ from the hidden layer to the input layer, the adjusting formula is

(17) $$w_{ij}\left(t+1\right)=w_{ij}\left(t\right)+\eta O_{j}^{{}^{\prime }}\left(1-O_{j}^{{}^{\prime }}\right)\delta_{k}w_{jk}O_{i}$$

In Equation (17), $\delta_{k}$ is the error in the output node *k*.

Step 5 Iterative loop. Return to Step 2 and iteratively operate until the error meets the requirements.

### 5.3. Parameters Setting

The feature vector will be used as input to the neural network. In this paper, the activity is classified according to which types of activity are taking place, the corresponding element will be set as 1, and the remaining elements will be zero. For instance, suppose there are five kinds of activities in total. If an unknown activity is classified as the second kind of activity, then the output value will be [0, 1, 0, 0, 0]. Therefore, the number of neurons in the output layer and the number of activity types will remain the same. Besides, the number of neurons in the hidden layer can make a great difference to the performance of the BP neural network. If the number of neurons is too few, the network cannot fully describe the relationship between input and output. On the contrary, if the number of neurons is too large, it will lead to the network needing to take a longer time to learn. What is worse, the classification results may turn out poorly. So, it is important to choose a suitable parameter as the number of neurons in the hidden layer. For a three-layer neural network, there are some common empirical formulas. The empirical formula selected in this paper is as follows:

(18) $$m=\sqrt{xy}$$

where *m* represents the number of neurons in the hidden layer, *x* represents the number of neurons in the input layer, and *y* represents the number of neurons in the output layer. The number obtained by this formula is usually not an integer, therefore, the number of neurons is selected from two integers that are near to the calculated results.

The neural network toolbox in *MATLAB* [53] is used to classify activities. The learning rate is set to 0.1. The Trainlm function is selected as the training function, which uses the Levenberg-Marquardt algorithm to update the weights. Moreover, it is the fastest back propagation algorithm in this toolbox. Because the output value of the neural network is 0 or 1, the hidden layer chooses the logsig function as the transfer function since it can map the input values on the [−∞,∞] interval to the [0,1] interval. In addition, the output layer selects the purelin function as the transfer function. The performance evaluation index is the mean square error, that is, reducing the mean square error to the acceptable range through iterative learning.

### 5.4. Feature Selection

As we mentioned in section4, a raw signal can be decomposed to several IMFs and their IA and IF can be obtained. It can be seen from the Figure 2 that the first IMF has the highest frequency and the penultimate signal has the lowest frequency. We regard the first two IMFs as high frequency noise. Usually, daily activities belong to the low frequency motion and the energy of them located at low frequency band [9]. Meanwhile, the number of IMFs from decomposition is a variable, in order to ensure these features can be obtained. The presented method considers the mean and variance of the third and the fourth IMFs as features to be selected. Then based on all IA and IF, the Hilbert spectrum and relevant physical quantities such as IE, MS, DS can be deduced. There are some relevant papers using EMD to do the feature extraction [9,10,11], but they only consider using the mean and variance of all or part of IA and IF. However, according to the above analysis from Figure 5, the IE can be an obvious feature for activity recognition. So based on their work, besides using the IA and IF, we also discuss the effect when the mean and variance of IE and MS as features.

## 6. Results

This section introduces how to use the features of HHT for activity recognition. Moreover, we discuss some different conditions upon experiment, including using each feature independently, combining some of them, and analyzing the effect of two scenarios: dependent subjects and independent subjects. In addition, we make some comparisons with relevant work.

### 6.1. Performance Measures

In order to compare our results with [54], this paper uses same performance measures: recall, precision, F-measure and accuracy, which are also commonly used in the field of classification. Firstly, we introduce the concept of confusion matrix, that is a matrix where each column of this matrix represents the activities in a annotated (or target) class while each row represents the activities in an recognized (or output) class. Moreover, we represent the element in the *i*th row and *j*th column as $P_{ij}$, the sum of all elements in the *i*th row as $S_{i}$, and the sum of all elements in the *j*th column as $R_{j}$. Then let *C* be the total number of activity classes and *N* be the total number of samples of all activities. Based on these notations, we can clearly define these performance measures as follows.

(19) $$recall=\frac{1}{C}\sum_{i=1}^{C}\frac{P_{ii}}{R_{i}}$$

(20) $$precision=\frac{1}{C}\sum_{i=1}^{C}\frac{P_{ii}}{S_{i}}$$

(21) $$F-measure=2\frac{precision\cdot recall}{precision+recall}$$

(22) $$accuracy=\frac{1}{N}\sum_{i=1}^{C}P_{ii}$$

### 6.2. Recognition Performance When Only Using IE as Feature

As described in the previous sections, this paper uses three wearable sensors to collect activity data. Then, these data are divided into several segments for every 5.12 s. Each sensor collects data from its x, y, and z axes. This paper classifies 12 activities which include most common activities in daily life. It is important, because we can provide useful guidelines to users if we can recognize these activities accurately.

We divide each subject’s activity data that have been preprocessed into two parts: one half for training the neural network and the other half for testing the training effect. For each data segment, we can get several eigenvalues. Then, we use training data to train the neural network and use testing data to evaluate the classification accuracy. According to Equation (18), the structure of neural network in this experiment is 18-14-12. The three numbers represent the number of neurons in the input layer, the hidden layer, and the output layer, respectively. The confusion matrix of training data is shown in Table 4.

Using the trained neural network to classify the data of the other parts, testing data, the confusion matrix is obtained in Table 5.

Based on the above two confusion matrices, Equations (20)–(23), we can obtain the performance measures for these two conditions in Table 6.

In our real life, however, only the untrained data needs to be classified, thus, the results of Table 5 are more practical and our focus is on trying to improve the performance measures of testing data. Therefore, in the next subsections, we will discuss the activity recognition from this perspective.

### 6.3. Recognition Performance of Other Features of HHT

Through the experiment, the effect of three different types of features on activity recognition is analyzed. The first feature is the mean and variance of IE, which we have discussed in Section 6.2. The second feature is the mean and variance of MS and the third one is the mean and the variance of IA and IF. We use the data coming from the accelerometers of three sensors. The accelerometer has three (*x*, *y*, *z*) axis, so when the IE or MS is considered as a feature for activity recognition, the number of eigenvalues is 18. Then, a raw signal can always be decomposed into several IMFs and the total number of IMFs is variable. Therefore, for each signal, the third and the fourth IMFs are selected and this the reason we have illustrated this in Section 5.4. Next, the IA and IF of each IMFs are extracted as features. Thus, when we use the IA and IF as feature, 72 eigenvalues can be obtained in total.

**Figure 10:**
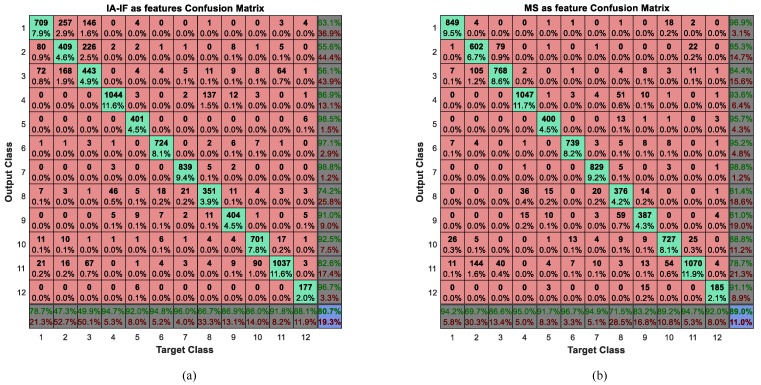
Confusion matrices of using MS, instantaneous amplitude-instantaneous frequency (IA-IF) as features (**a**) IA-IF as features; (**b**) MS as feature. The diagonal cells (green cells) show the number and percentage of correct classification. The red cells show where the classifier has made mistakes. The dark cells in the bottom row show the accuracy of each annotated class, while the dark cells in the right column show the accuracy of each recognized class. The purple cell in the bottom right of the matrix shows the overall accuracy.

In this experiment, the structure of a neural network is 18-14-12 when the IE or MS was considered as a feature for activity recognition. If the features are IF and IA, the structure of the neural network is 72-29-12. The three numbers represent the number of neurons in the input layer, the hidden layer, and the output layer respectively. The confusion matrices obtained by using MS, IA and IF as features are shown in Figure 10.

As we can seen from Figure 10, the judge error between the second activity sitting and the third activity standing is huge, especially when using IA and IF as features, the correct rates are less than 50%. Maybe this is the reason that these two activities be seen as the same activity in the [54]. Meanwhile, we have discussed these activities in Figure 5. The features of them are very similar, which leads us to conclude that it is difficult to distinguish them. In addition, from the results of the confusion matrix, we can intuitively draw the conclusion that the effect of IA and IF is much worse than IE or MS in the activity recognition. To compare and evaluate these three types of features, we calculate their performance measures, as shown in Table 7.

As we predicted, the results shows that the effect of IE and MS are roughly equivalent. The precision and accuracy of IE are a little better than that of MS while the recall and F-measure of MS are relatively better than that of IE. From the previous analysis, the figures of the MS among different activities sometimes looks very similar, nevertheless, we know that, according to the classification results, the neural network can still clearly find these differences and classify them correctly. However, when the IF and IA are considered as features for activity recognition, the performance measures are rather poor. The error rate of some activities is very high, and it is difficult to apply it to real life. We maintain that the IF and IA are instantaneous features, which describe the characteristics of a signal at a certain time, but there are too many eigenvalues to put them all into classification. Thus, we use the mean and variance to process these eigenvalues and use them as inputs to the BP neural network. However, this process eliminates the instantaneous feature and leads to steps which aim to stop the instantaneous feature becoming meaningless. This results in the effect of IA and IF being unsatisfactory. It will be better if we put all instantaneous features into classification without considering time consumption and network magnitude, but it is difficult to process hundreds of, and even thousands of eigenvalues. However, for the IA and IF, the correct rates of some activities like cycling are higher than the other two methods. Therefore, we infer that this method may have a special characteristic which can help improve the performance measures of the other two methods and we consider combining these features in the next subsection.

### 6.4. Recognition Performance of Multi-Features Combination

**Figure 11:**
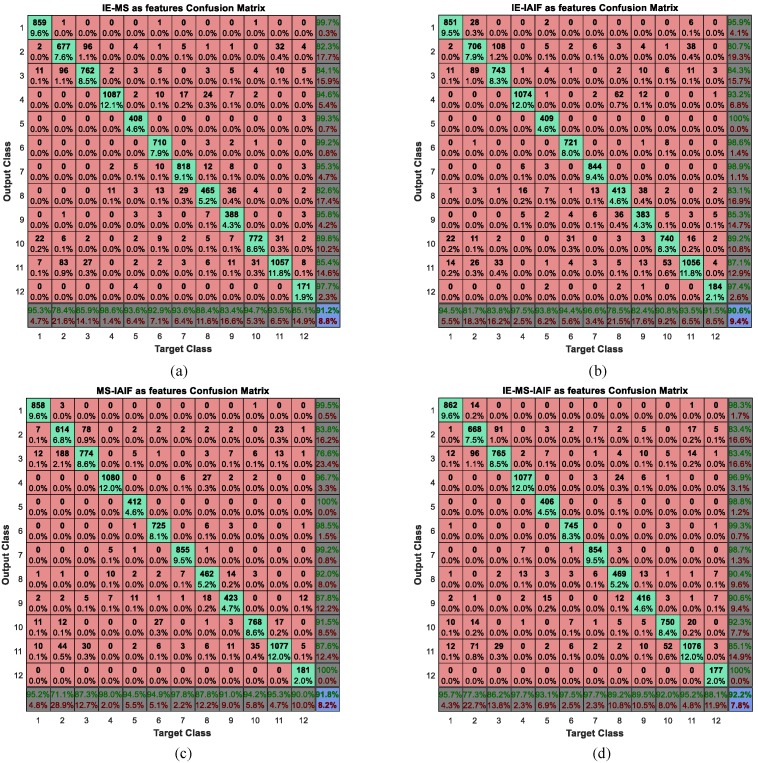
Confusion matrices of multi-features combinations. (**a**) IE and MS as features; (**b**) IE and IA-IF as features; (**c**) MS and IA-IF as features; (**d**) IE, MS, and IA-IF as features. The diagonal cells (green cells) show the number and percentage of correct classification. The red cells show where the classifier has made mistakes. The dark cells in the bottom row show the accuracy of each annotated class, while the dark cells in the right column show the accuracy of each recognized class. The purple cell in the bottom right of the matrix shows the overall accuracy.

According to the analysis of above subsection, we can get relatively satisfied recognition results when using IE or MS as features. In order to further improve the performance of activity recognition, we consider combining these features both pairwise and by combing all four. Thus, we will have four different types of feature groups including IE-MS, IE-IAIF, MS-IAIF, and IE-MS-IAIF. Then, we take these feature groups as inputs to the BP neural network. The corresponding confusion matrix of each type of feature group can be gained. In this experiment, the structure of the neural network is 90-32-12 when the IE-IAIF or MS-IAIF was considered as a feature for activity recognition. If the features are the IE-MS, the structure of the neural network is 36-20-12 and if the features are the IE-MS-IAIF, the structure of the neural network is 108-36-12. The three numbers represent the number of neurons in the input layer, the hidden layer, and the output layer respectively. We plot their confusion matrices in Figure 11.

**Figure 12:**
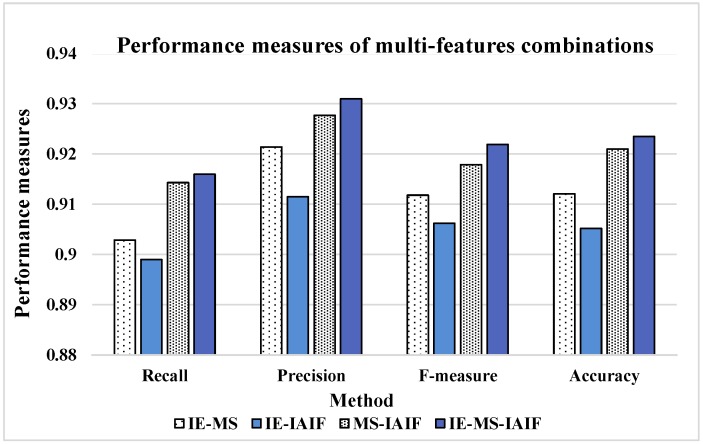
Performance measures of multi-features combinations.

To intuitively compare the effect of these feature groups, we calculate their performance measures and show the results in Figure 12.

It can be seen from the results that nearly all the numbers in Figure 12 are bigger than 0.9 while all the numbers in Table 7 are less than 0.9, so the performance of each feature group is better than that of an individual feature. Thus, we believe that multi-features combination can further improve the performance of activity recognition. In addition, when we use each feature separately, the performance measures of IE and MS are much better than that of IAIF. However, when we combine these features in pairs, the MS-IAIF rather than IE-MS shows the best performance. We think that IE and MS may contain many similar characteristics which mean that it is difficult for these two features to play a complementary role for each other. At the same time, according to the above analysis, the correct rate of some activities using IAIF as a feature is better than the condition of using MS. Thus, IAIF can provide some special characteristics for MS to improve the performance. Moreover, it shows the best results when we combine all three features together, but it only a little bit better than the results of MS-IAIF.

### 6.5. Recognition Performance of Independent Subject

In the above discussion, each type of activity data is composed of all subjects’ corresponding data and we divide these data into two parts: training data and testing data. However, in our daily life, people focus more on pursuing personalized experience and use the tailor-made devices. Thus, the independent subject validation is important for the activity recognition system. In this subsection, the effect of the presented method in the scenario of independent subject is analyzed. We use the same data preprocessing method as in the scenario of the dependent subject and process each subject’s data independently. That is to say, we will separately divide each subject’s data into two parts without mixing them with any other subjects’ data. According to the analysis of the previous subsection, the feature IE-MS-IAIF shows the best performance in the scenario of dependent subject; therefore, we also use this feature as an example to do the experiment. Besides, in this dataset, only five subjects (including subject ID 1, 2, 5, 6, and 8 ) finish all 12 activities that we want to classify. All other datasets have some missed activity type that mean they cannot be used. As for the data of subject ID 6, the duration of its activity, rope jumping, is less than 3 s, which is too short to use. Thus, in this experiment, we have data from four subjects and we show the results of them in Table 8.

In order to integrate their respective results into an overall performance measures we propose the definition of overall performance measures which is denoted by

(23) $$overall-PM=\sum_{i=1}^{m}\frac{N_{i}}{N}PM_{i}$$

(24) $$overall-PM=\left[\begin{array}{c} overall-recall\\ overall-precision\\ overall-F-measure\\ overall-accuracy \end{array}\right]$$

(25) $$PM_{i}=\left[\begin{array}{c} recall_{i}\\ precision_{i}\\ F-measure_{i}\\ accuracy_{i} \end{array}\right]$$

where overall-PM represents overall performance measures that are composed of four specific indexes, $PM_{i}$ is performance measures of the *i*th subject that is also composed of four indexes, and they are illustrated in Equations (24) and (25). In addition, *m* is the total number of subjects, which is 4 in this experiment, $N_{i}$ is the sample number of *i*th subject, and *N* is the total number of all samples. Then, we can get the complete results of this experiment in Table 8.

As we can see from the Table 8, performance measures differ from individual to individual because everyone has their own behaviour characteristic. Some people like subject ID 2 may make a big move during an action, which makes each activity can be easily distinguished, while some people like subject ID 5 may make smaller movements, which introduces difficulties to activity recognition. This is the challenge that we face in our daily life. Besides, the more important reason for this phenomenon is that the duration of some of subject ID 5’s activities is too short. This results in the sample size of these activities being small and affects the classification performance of the BP neural network. However, it is not a problem for practical application because the user can supply enough activity data for the algorithm. Thus, we can focus more on the results of the other three subjects. An overall-PM2 is calculated which does not concern the result of subject ID 5. All four performance measures of Overall-PM1 are bigger than 0.91 while those of Overall-PM2 are bigger than 0.93. In the meantime, as for the sample size, although the duration of some activities of subject ID 5 are only about 1 min, the four indexes can be satisfied. For any subject, most of the activities’ durations are less than 3 min, which is short. Nevertheless, the results show relatively high performance. Thus, the presented method can realize accurate activity recognition with a small amount of training data and we can believe that if we can give enough data as inputs, the results can be further improved.

### 6.6. Comparing with Relevant Work

Many related works [29,30,31,32,33,34,35,36,37] discuss the classification methods for activity recognition. In these papers, several classification approaches are introduced at the same time. Then, the author can determine a relatively good method according to some specific evaluation indexes. In addition, we used fuzzy classification [55] and the FFT algorithm to process other datasets before, and good classification results are gained with the proposed methods. Moreover, the dataset we use in this paper is from the experiment of [54]. In the work [54], the authors propose both dataset and benchmark for different methods of human activity recognition. Therefore, in order to evaluate our work, we make a comparison of the classification results with the outcome presented in [54]. In that paper, independent subject evaluation is done with leave-one-subject-out (LOSO) 9-fold cross-validation and dependent subject is done with standard 9-fold cross-validation. According to the previous analysis, the scenario of an independent subject is more common and practical, which allows a device to be more suitable for a specific user. Thus, we compare our results of the independent subject in Table 8 with the results from [54] which uses LOSO 9-fold cross-validation. Meanwhile, the activity recognition experiment we undertook is described as all the activity recognition tasks in that paper, so the corresponding results of the task are selected.

**Figure 13:**
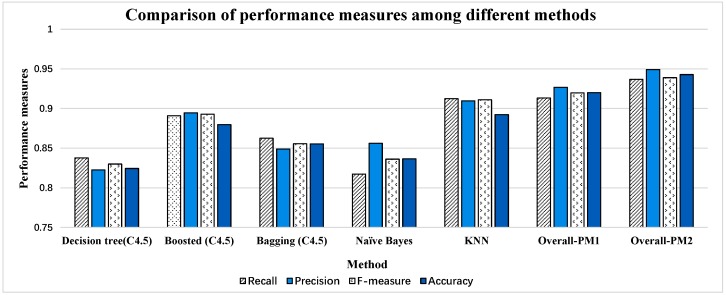
Comparison of performance measures among different methods. The results of the first five methods all come from [54] with leave-one-subject-out (LOSO) 9-fold cross-validation. Overall-PM1 and Overall-PM2 are the results of proposed method in Section 6.5.

It can be seen form the Figure 13 that the performance measures of the first four methods are all less than 0.9. KNN shows the best results in [54] which has a similar effect (slightly lower) to the results of Overall-PM1. If we do not consider subject ID 5 because of the sample size, Overall-PM2 has outperformed KNN significantly. There are some differences between the ways we preprocess the dataset. Besides, different classifiers are used to classify activities. However, the effectiveness of the HHT is remarkable. Especially, when the processed data in activity dataset are nonlinear and non-stationary (it can be seen in Figure 8), and HHT has obtained a very good effect processing this type of data in other areas [22,23,24,25,26,27,28]. Hence, the proposed method is verified to be valuable and practical in activity recognition and it can effectively make up the shortcoming of traditional methods when processing activity data.

## 7. Limitation and Future Work

Before we summarize the paper, current limitations of the proposed method should be pointed out to direct our future works.

Firstly, although the PAMAP2 dataset we used in this paper is well known for activity recognition and benchmarking, the dataset still contains null values and missing data fields due to packet loss in signal collection or the misuse of sensors by practitioners. To the best of our knowledge, how to deal with missing data is another critical issue for activity data processing based on body-worn sensors. In this paper, we only conduct a set of simple dropping policies to deal with the problem. For example, if there are missing data from one sensor in a certain time point, we will drop all data which are collected from three sensors in this time point. These policies are simple to facilitate our experimental study on feature extraction. Nevertheless, our means for missing data will break the continuity of activity data, or may reduce the recognition accuracy. Accordingly, in our future works, we plan to evaluate the influence of missing data to feature extraction for activity recognition, and to investigate some useful methods like interpolation or prediction, with the objective to fix the problem of missing data and improve recognition effect.

Another limitation of our work is that, due to the limitation of time and space, we do not conduct a detailed study on the impact of sensor numbers on the proposed feature extraction approach. In the view of beauty and comfort considerations, however, people are inclined to wear fewer sensors. Thus, it is important to explore the relationship between recognition results and the number of sensors. Moreover, we can find a tradeoff between them based on the relationship. We will also investigate a possible future research task, to integrate other various classification tools (beyond BP neural network) with the proposed feature extraction methods. As we mentioned in Section 2.2, many kinds of classifiers are widely used in activity recognition. It is valuable to study the effects of the proposed methods with different classifiers.

## 8. Conclusions

In this paper, a feature extraction method based on HHT has been proposed for activity recognition since the activity data are non-stationary and nonlinear. The experimental results show that our approach is capable of handling this problem and three conclusions can be drawn.

Firstly, In addition to the features (IA and IF) from EMD, we also use two other related features (IE and MS) from Hilbert spectral analysis. In the scenario of dependent subjects, the effect of multi-features combination and single-feature are discussed. The comparison results show further performance improvement with multi-features combination. At the same time, the highest performance measures can be obtained when we combine all three features together, that is, using IE-MS-IAIF as features.

Secondly, in the scenario of the independent subject, the four overall performance indexes: recall, precision, F-measure, and accuracy can reach 0.9337, 0.9417, 0.9353, and 0.9377 respectively. The experimental results from two scenarios show that our method is both suitable for the scenario of independent subject and dependent subject.

Finally, compared with relevant paper, the relatively high performance of the presented method demonstrated that the effectiveness of the HHT is remarkable and it can make up the shortcoming of traditional methods when the processed data are nonlinear and non-stationary.

## Figures and Tables

**Table 1:** Activities performed by subjects.

Number	Sum (in Seconds)	Number	Sum (in Seconds)
1-lying	1925.15	11-car driving	545.18
2-sitting	1851.80	12-ascending stairs	1172.00
3-standing	1899.23	13-descending stairs	1049.27
4-walking	2387.53	16-vacuum cleaning	1753.45
5-running	981.92	17-ironing	2386.82
6-cycling	1645.93	18-folding laundry	998.74
7-nordic walking	1881.00	19-house cleaning	1871.83
9-watching TV	836.45	20-playing soccer	469.12
10-computer work	3099.31	24-rope jumping	493.54

**Table 2:** The meaning of each column in dataset.

Number	Meaning	Number	Meaning
1	timestamps (s)	4-20	IMU ${}^{3}$ wrist
2	activity ID ${}^{1}$	21-37	IMU chest
3	heart rate (bpm ${}^{2}$)	38-54	IMU ankle

^1^ ID: identification; ^2^ bpm: beat per minute; ^3^ IMU: inertial measurement unit.

**Table 3:** The meaning of each column in IMU data.

Number	Meaning
1	temperature (${}^{\circ }$C)
2–4	3D-acceleration data (ms${}^{-2}$), scale: ±16 g, resolution: 13-bit
5–7	3D-acceleration data (ms${}^{-2}$), scale: ±6 g, resolution: 13-bit
8–10	3D-gyroscope data (rads${}^{-1}$)
11–13	3D-magnetometer data (μT)
14–17	orientation (invalid in this data collection)

**Table 4:** Confusion matrix of training data with three sensors using instantaneous energy density (IE) as feature.

Recognized Activity	Annotated Activity											
	1	2	3	4	5	6	7	8	9	10	11	12
**1**	901	0	0	0	0	0	0	0	0	4	0	0
**2**	0	821	6	0	0	4	0	0	0	5	31	0
**3**	0	38	791	0	0	0	0	0	0	7	54	0
**4**	1	0	0	1082	0	0	12	3	8	0	0	0
**5**	0	0	0	0	441	0	0	0	0	0	0	0
**6**	1	0	0	1	0	720	2	0	0	33	11	0
**7**	0	0	0	5	1	0	872	0	0	0	0	0
**8**	0	6	22	76	0	2	13	337	45	10	17	0
**9**	0	5	21	29	0	5	16	31	341	8	10	1
**10**	0	0	2	1	0	16	5	9	2	755	28	0
**11**	0	10	18	0	0	8	0	0	0	16	1084	0
**12**	0	0	0	0	0	0	0	5	0	0	0	199

The digits map one to one with activities; 1-Lying, 2-Sitting, 3-Standing, 4-Walking, 5-Running, 6-Cycling, 7-Nordic walking, 8-Ascending stairs, 9-Descending stairs, 10-Vacuum cleaning, 11-Ironing, 12-Rope Jumping.

**Table 5:** Confusion matrix of testing data with three sensors using IE as feature.

Recognized Activity	Annotated Activity											
	1	2	3	4	5	6	7	8	9	10	11	12
**1**	848	0	7	0	0	7	0	0	1	26	12	0
**2**	0	663	85	0	0	12	2	0	0	5	97	0
**3**	0	87	752	0	0	1	0	0	0	6	41	0
**4**	0	1	1	1073	0	0	14	11	2	0	0	0
**5**	0	3	0	0	410	0	7	6	1	3	3	3
**6**	2	0	1	20	0	688	10	6	0	21	16	0
**7**	0	2	0	12	0	3	841	12	0	0	4	0
**8**	0	0	2	74	0	2	17	399	23	6	3	0
**9**	0	0	6	16	10	9	9	28	358	17	10	2
**10**	0	3	10	0	0	17	6	11	2	701	65	0
**11**	0	29	12	0	0	4	3	0	0	36	1046	0
**12**	0	0	2	1	4	0	2	2	4	0	2	184

The digits map one to one with activities; 1-Lying, 2-Sitting, 3-Standing, 4-Walking, 5-Running, 6-Cycling, 7-Nordic walking, 8-Ascending stairs, 9-Descending stairs, 10-Vacuum cleaning, 11-Ironing, 12-Rope Jumping.

**Table 6:** Performance measures when only using IE as feature.

	Performance Measures	Recall	Precision	F-Measure	Accuracy
Data Type					
**Testing data**		0.9135	0.9305	0.9263	0.9219
**Training data**		0.8802	0.8998	0.8882	0.8899

**Table 7:** Performance measures when using different features of Hilbert-Huang transform (HHT).

	Performance Measures	Recall	Precision	F-Measure	Accuracy
Feature Type					
**IE**		0.8802	0.8998	0.8882	0.8899
**MS**		0.8829	0.8924	0.8900	0.8876
**IAIF**		0.8107	0.8276	0.8075	0.8191

**Table 8:** Performance measures in the scenario of independent subject.

	Performance Measures	Recall	Precision	F-Measure	Accuracy
Type					
**Subject ID 1**		0.9252	0.9378	0.9191	0.9314
**Subject ID 2**		0.9469	0.9562	0.9512	0.9516
**Subject ID 5**		0.8468	0.8634	0.8650	0.8550
**Subject ID 8**		0.9376	0.9528	0.9455	0.9451
**Overall-PM1 ${}^{1}$**		0.9133	0.9268	0.9197	0.9200
**Overall-PM2 ${}^{2}$**		0.9368	0.9491	0.9389	0.9429

${}^{1}$ Overall-PM1 is the overall performance measures which integrate the results of all subjects; ${}^{2}$ Overall-PM2 is the overall performance measures which do not concern about subject ID 5.
